# The prevalence of malocclusion and the need for orthodontic treatment among adolescents in the northern border region of Saudi Arabia: an epidemiological study

**DOI:** 10.1186/s12903-018-0476-8

**Published:** 2018-02-02

**Authors:** Ravi Kumar Gudipaneni, Raed F. Aldahmeshi, Santosh R. Patil, Mohammad Khursheed Alam

**Affiliations:** 1 Department of Pedodontics, College of Dentistry, Aljouf University, Sakaka, Kingdom of Saudi Arabia; 2 College of Dentistry, Aljouf University, Sakaka, Kingdom of Saudi Arabia; 3 Department of Oral Medicine and Radiology, College of Dentistry, Aljouf University, Sakaka, Kingdom of Saudi Arabia; 4 Orthodontic Department, College of Dentistry, Aljouf University, Sakaka, Kingdom of Saudi Arabia

**Keywords:** DHC, IOTN index, Malocclusion, Prevalence

## Abstract

**Background:**

To assess the prevalence of malocclusion and orthodontic treatment need among adolescents using the dental health component (DHC) of the index of orthodontic treatment need (IOTN).

**Methods:**

A descriptive cross-sectional study was conducted among 500 (mean age 16.25 ± 1.09) adolescents randomly selected from the northern border region of Saudi Arabia (KSA). The northern border region is sub-divided into three governorates: Ar’ar (186), Rafha (142) and Turayf (172). The data were recorded in questionnaires to assess the prevalence of malocclusion and estimate of DHC of the IOTN index.

**Results:**

The most common malocclusions in order of prevalence were Angle’s Class I (52.8%), Angle’s Class II (31.8%), Angle’s Class III (15.4%), crowding (47.2%), excessive overjet (> 2 mm) (22.2%), reduced overjet (< 1 mm) (11.4%), excessive overbite (> 2 mm) (23.4%), reduced overbite (< 1 mm) (12.2%), anterior crossbite (4.8%), posterior crossbite (9.4%) and open bite (4.6%). The most common facial profiles determined in the sagittal plane, were the straight facial profile (49.2%), convex (42.6%) and concave (8.2%). The prevalence of Grade 1 and 2 DHC was 49.4%, Grade 3 was 29.6%, Grade 4 and 5 was 21%. The grades of DHC of IOTN index were as follows: 48.73% of males and 50.22% of females showed grades 1 and 2. Grade 3 was observed in 30.32% of males and 28.69% of females. Grades 4 and 5 were recorded in 20.93% of males and 21.07% of females.

**Conclusions:**

The prevalence of malocclusion and orthodontic treatment need among the north border region of KSA is comparable with that of other regional studies.

## Background

The prevalence of malocclusion has been reported to vary from nation to nation and among diverse gender and age groups. Previously, a number of investigators reported the prevalence of malocclusion in Saudi populations [1–3]. All of these studies were carried out in the central region and in the eastern region of Saudi Arabia. No representative data on the prevalence of dentofacial characteristics are available in the northern border region of KSA, and there has been a steady increase in the number of Saudi adolescent patients seeking orthodontic treatment has increased markedly during recent years. A scarcity of published reports on the distribution of the malocclusion traits among adolescent age groups in Saudi populations provided the rationale for the current investigation. Therefore it is important to have data on malocclusion in order to estimate the total need for treatment. As per the authors’ best knowledge, this will be the first prevalence study of malocclusion from the northern border region of KSA. The present study was therefore designed to determine the prevalence of malocclusion in adolescents in the northern border region of Saudi Arabia and to assess the DHC of the orthodontic treatment need index, a time-tested index that is used internationally. Shaw et al. [4]. formulated the IOTN in the U.K, because of its simplicity and convenience it is widely accepted and perceived as a system to assess treatment need [5–7]. The legitimacy and unwavering quality of the IOTN index have been widely verified in diverse nations by various researchers [4, 8–10]. Epidemiological studies related to malocclusion not only help in orthodontic treatment planning but also offer a legitimate exploration tool for recognizing the environmental and hereditary elements in the etiology of malocclusion [11]. Furthermore, these studies will aid in comprehending the required assets and preventive measures and in planning the proper health care programmes.

The present study has been under taken to assess the prevalence of malocclusion and assess the orthodontic treatment needs according to DHC of IOTN among adolescents among adolescents residing in the northern border region of the Kingdom of Saudi Arabia.

## Methods

### Study population

This study was conducted in the northern border region of Saudi Arabia. The northern border region is sub-divided into three governorates: 'Ar'arRafha and Turayf. Its capital is 'Ar'ar. It has an area of 111,797 km^2^ and a population of 320,524. Sample size was calculated based on the prevalence of malocclusion in a pilot study (*p* = 23%). A total sample size of 500 individuals will be sufficient to detect statistically significant difference of 5% with 95% confidence interval and 80% power using chi square test and considering design effect of two. Sample for each region was decided by proportionately, depending on percentage of total population it represents. The present study group consisted of 500 Saudi adolescents, male and female, aged from 14 to 18 years, who attended the various ministry of health hospitals in Ar’ar (186), Rafha (142), and Turayf (172). In this study, subjects were selected using random sampling procedures. The study was conducted over a period of six months from March 2015 to August 2015. The adolescents who had or who were having orthodontic treatment and systemic health problems, developmental anomalies, such as ectodermal dysplasia, cleft lip or palate, and down syndrome, were excluded from the study. The study protocol was reviewed and ethical clearance was obtained before the start of the study from the Ethical approval Committee of College of Dentistry, Aljouf University, KSA [No: COD15–16/00217]. An official permission was obtained from the concerned authorities of ministry of health hospitals. The parent or guardian of the child provided the consent on behalf of the patient.

### Examination procedure

All the subjects were examined by a single examiner after obtaining the informed consent from the subjects and their parents prior to clinical examination. Before the survey, the examiner an intern dentist participated in a training and clinical calibration program under supervision of department of pediatric dentistry. Following this training, 10% of the adolescents were re-examined by the investigator after a period of two weeks to assess intraexaminer reliability. The kappa statistic is a measure of agreement that has been corrected for chance agreement, so the intrarater reliability was assessed with Kappa statistics (0.82). A well trained recording clerk assisted the examiner throughout the oral examination procedure. The examination was performed in a dental chair under artificial illumination and the malocclusion was recorded according as described by WHO, Oral health Survey, basic methods by using Community Periodontal Index [CPI] probe and plane mouth mirror [12]. Each subject was examined for the type of malocclusion, including Angle’s classification, open bite, crossbite, overjet, overbite, crowding, spacing as defined in Table 1, Facial profile was determined in the sagittal plane and was assessed as straight, concave or convex depending on the spatial relationship or harmony between mandible and maxilla.

**Table 1:** Variables and detentions used in the study

Antero-posterior deviations	
Angle’s molar relationship	Class I
	Class II
	Class III
Overjet	Normal (1–2 mm),
	Excess (> 2 mm),
	Reduced (< 1 mm)
	Reverse overjet or Anterior crossbite (< 0)
Vertical deviations	
Overbite	Normal (1–2 mm)
	Excess (> 2 mm),
	Reduced (< 1 mm)
Open bite	Absent
	Present
Transverse deviation	
Posterior crossbite	Absent
	Present
Alignment	
Spacing	Absent
	Present
Crowding (Overlapping of erupted teeth due to insufficient space or lack of space for teeth to erupt in the dental arch)	Absent
	Present
Facial profile	
Type	Straight profile
	Convex profile
	Concave profile

The grades of orthodontic treatment using DHC of IOTN index were recorded. The DHC records the various occlusal traits of malocclusion and the treatment needs of the subjects are assorted as grade 1 (no treatment need), grade 2 (mild need), grade 3 (moderate need), grade 4 (severe need) and grade 5 (extreme need). A survey proforma was used for data recording and prepared according to the guidelines of WHO Oral Health Assessment [12]. The data were recorded in the questionnaires.

Statistical analysisThe recorded data were transferred from survey proforma to an SPSS (Version 22, SPSS Inc., Chicago, USA). The chi-square test and Z-proportionality test were applied and the significance level was set at 0.05 (*P* < 0.05).

## Results

A total of 500 adolescents met the inclusion criteria. Of these, 277 were males (55.4%) and 223 were females (44.6%).

As shown in Table 2, out of 500 patients:

- Two hundred and fourty six (49.2%) of patients had a straight profile, while 213 (42.6%) of patients had convex profile and 41 (8.2%) of patients had a reduced profile. The difference was found to be significant (*p* < 0.001).
- Two hundred and sixty four (52.8%) of patients had Angle’s Class 1 occlusion, 159 (31.8%) had Angle’s Class II and 77 (15.4%) had Angle’s Class III occlusion. The difference was found to be significant (*p* < 0.001).
- Three hundred and thirty two (66.4%) of patients had normal overjet, 111 (22.2%) of patients had excessive overjet and 57 (11.4%) of patients had reduced overjet. The difference was found to be significant (*p* < 0.001).
- Three hundred and twenty two (64.4%) of patients had normal overbite, 117 (23.4%) of patients had excessive overbite and 61 (12.2%) of patients have reduced overbite. The difference was found to be significant (*p* < 0.001).
- Crowding was present in 47.2% of patients, while 52.8% of patients had no crowding. The difference was not found to be significant (*p* = 0.8944).
- Spacing was present in 27.2% of patients, while 72.8% of patients had no spacing. The difference was found to be significant (*p* < 0.001).
- An anterior crossbite was present in only 4.8% of patients, while 95.2% of patients had no anterior crossbite. The difference was found to be significant (*p* < 0.001).
- A posterior crossbite was present in only 9.4% of patients, while 90.6% of patients had no posterior crossbite. The difference was found to be significant (*p* < 0.001).
- An open bite was present in only 4.6% of patients, while 95.4% of patients had no open bite. The difference was found to be significant *(p* < 0.001).

**Table 2:** Prevalence of different malocclusions and occlusal traits in the studied sample

Angle’s malocclusion	N (%)	*P*-value (Chi-Square)
Occlusal traits		
Angle’s Class 1	264 (52.8%)	< 0.001
Angle’s Class 2	159 (31.8%)	
Angle’s Class 3	77 (15.4%)	
Overjet		
Normal	332 (66.4%)	< 0.001
Excessive	111 (22.2%)	
Reduced	57 (11.4%)	
Overbite		
Normal	322 (64.4%)	< 0.001
Excessive	117 (23.4%)	
Reduced	61 (12.2%)	
Crowding		
Present	236 (47.2%)	0.8944
Absent	264 (52.8%)	
Spacing		
Present	136 (27.2%)	< 0.001
Absent	364 (72.8%)	
Anterior crossbite		
Present	24 (4.8%)	< 0.001
Absent	476 (95.2%)	
Posterior crossbite		
Present	47 (9.4%)	< 0.001
Absent	453 (90.6%)	
Open bite		
Present	23 (4.6%)	< 0.001
Absent	477 (95.4%)	
Facial Profile		
Straight	246 (49.2%)	< 0.001
Convex	213 (42.6%)	
Concave	41 (8.2%)	

As shown in Table 3, out of 500 patients in whom 247 (49.4%) had no need for any treatment, 148 (29.6%) needed a borderline treatment and 105 (21.0%) needed a definite treatment. Furthermore, of the 247 patients (49.4%) who did need any treatment, 48.73% were males and 50.22% were females. Similarly, of the 148 patients (29.6%) who needed borderline treatment, 30.32% were males and 28.69% were females. Of the 105 patients (21.0%) who needed definite treatment, 20.93% were males and 21.07% were females. The association between the genders was not found to be statistically significant (chi-square = 0.1672, *p* = 0.9201).

**Table 3:** Dental health components of IOTN grades

DHC Grade	Need for Treatment	Males	Females	Total	Percentage	Chi-square	*P*-value
Grade 1 and 2	No need for treatment	135(48.73%)	112(50.22%)	247	49.4%	0.1672	0.9201
Grade 3	Borderline	84(30.32%)	64(28.69%)	148	29.6%		
Grade 4 and 5	Definite treatment	58(20.93%)	47(21.07%)	105	21%		

## Discussion

This is the first study to provide knowledge about the prevalence of malocclusion in the northern border region of Saudi Arabia. Various studies have been reported in the literature from various countries describing the prevalence and types of malocclusion in adolescent age groups. In spite of this, comparisons of the observation from these studies are cumbersome because of the variations in the age and size of the study samples and the methodology adapted to record occlusal relationships. The prevalence of malocclusion is reported to vary by country, age and sex. Very few studies have been conducted in Saudi Arabia to assess the prevalence among adolescents [1–3]. The determination of the total frequency of malocclusion and the need for orthodontic treatment was considered one of the simplest methods of recording malocclusion, and this was the method used in our study. In the present study, we observed that 52.8% had Angle’s Class I occlusion, 31.8% had Class II and 15.4% had Class III. The prevalence of Angle’s Class I occlusion was higher and the prevalence of Angle’s Class III occlusion was lower. The findings of our results confirmed that the most prevalent malocclusion was Angle’s Class I followed by Angle’s Class II, while the least prevalent malocclusion was Class III (15.4%). Only a true class III molar relationship was recorded in this study. These results were in agreement with previous Saudi studies [13, 14]. Al-Emran et al. and Al-Balkhi and Zahrani reported that the most common type of malocclusion in the Saudi population was Class I followed by Class II division 1 and Class III [13, 14]. Abdullah MA reported that the most common dental malocclusion in Saudis was Angle’s Class I followed by the asymmetric molar relationship [3]. In Nigeria (76.5%) and Turkey (74%), a higher percentage of Angle’s Class I malocclusion was observed, while in Pakistan, a higher percentage of Angle’s Class II malocclusion was found among orthodontic patients (70.5%) [15–17]. A study done in urban Iranian adolescents by Borzabadi-Farahani A et al., the prevalence of Class I, Class II division 1, Class II division 2, and Class III malocclusions was 41.8%, 24.1%, 3.4%, and 7.8% respectively. Symmetric molar relationship was observed in 69.5% [18]. Elham SJ et al. reported the prevalence of malocclusion in 16-year-old Jordanian school children. They found that Class I was the most prevalent (55.3%), followed by Class II (17.5%) and Class III (1.4%) [19]. In a Turkish study, Angle’s Class I malocclusion was found in 64% of the study sample. The frequencies of Class II division 1 and Class II division 2 malocclusions were 19% and 5%, respectively. Class III malocclusion was present in 12% of the patients. These results were close to our observations [16, 19, 20]. The prevalence of increased overjet in our study was 22.2% and that of reduced overjet was 11.4%. These results were close to those of other Saudi studies [20]. Elham SJ et al. reported 24.7% increased overjet in 13–15-year-old Jordanian school children, which was close to the findings in our study [19]. Borzabadi-Farahani A et al. reported that an over jet of at least 3.5 mm or more was present in 28.1%; an overjet of more than 6 mm in 3.6%, and 4.2% had a reverse overjet in Iranian adolescents [18]. In the present study, 23.4% of subjects showed increased deep bite, 12.2% showed reduced overbite and 64.4% subjects showed normal bite. Asiry MA conducted a study on 1825 Saudi school children and reported excessive overbite in 6.68% of the study population [20]. Borzabadi-Farahani A et al. observed normal overbite was observed in 60.4%, while 34.5% had an increased and 2.2% a very deep overbite [18]. In the present study, crowding was observed in 47.2% of the study population, and this finding was close to those of the other studies [19, 20]. Generalized spacing was observed in 27.2% of subjects in our study. The space anomalies detected in Saudi adolescents of our study were similar to those reported by a previous Saudi study [20]. The anterior crossbite was observed in 4.8% of subjects and the posterior crossbite was observed in only 9.4% of subjects. The study by Elham SJ et al. reported that 1.9% of 13–15-year-old Jordanian schoolchildren had anterior crossbite and 7.1% had posterior crossbite [19]. Al-Balkhi and Zahrani reported that the prevalence of crossbites in Saudi patients was relatively high and almost equally distributed between the anterior and posterior regions of the dental arch [14]. The presence of open bite was found in 4.6% of subjects. Our results were close to the study performed by Elham SJ et al. and Asiry MA [19, 20]. We followed the procedure to assess the subjects sagittal plane and in sitting upright with the Frankfort Horizontal Plane parallel to the floor [21]. We found, a straight, convex and concave profile in 49.2%, 42.6% and 8.2% respectively.

In the present study, the results of the DHC of the IOTN index showed that only 21% of all subjects (20.93% males, 21.07% females) were in severe and extreme need of treatment (IOTN grades 4 and 5), whereas 29.6% of subjects (30.32% males, 28.69% females) were in grade 3, (borderline) and 49.4% of subjects were found to be below grade 1 and 2 (48.73% males, 50.22% females); these results were in line with other studies [22, 23]. Al-Azemi R et al. [23] reported that approximately 30% of adolescent Kuwaiti females had a definitive orthodontic treatment need (Grades 4 and 5). Hedayati Z et al. reported that 18.39% of an Iranian population showed severe and very severe need for treatment; 25.8% were borderline, while 48.1% had a slight need and 7.63% had no need for treatment [24]. Similiarly, Borzabadi-Farahani A et al. in 2009 done a study on Iranian school children and reported that 36.1% had definite need for orthodontic treatment, 20.2% borderline need and 43.8% showed slight or no need for treatment [25].

However, IOTN index have a role in the epidemiology and can be used for resource planning, but their predictive value to detect the future objective functional deficits or oral health problems is questionable. IOTN index will need revalidation over time with emerging research findings [26]. The results of the present study suggest lack of awareness among the adolescent about the severity of their existing malocclusion. This may be attributed to their weak oral health knowledge and parent’s negligence toward malocclusion.

## Conclusion

The prevalence of malocclusion and orthodontic treatment need among the north border region of KSA is comparable with that of other regional studies.

## Abbreviations

DHC: Dental health component
IOTN: ICC Intraclass correlation coefficient index of orthodontic treatment needs
KSA: Saudi Arabia
